# Bodipy Dimer for Enhancing Triplet-Triplet Annihilation Upconversion Performance

**DOI:** 10.3390/molecules28145474

**Published:** 2023-07-18

**Authors:** Min Gao, Le Zeng, Linhan Jiang, Mingyu Zhang, Yong Chen, Ling Huang

**Affiliations:** 1 Jiangxi Key Laboratory for Microscale Interdisciplinary Study, Institute for Advanced Study, Nanchang University, Nanchang 330031, China; yaogao@email.ncu.edu.cn; 2 Research Center for Analytical Sciences and Tianjin Key Laboratory of Biosensing and Molecular Recognition, Haihe Laboratory of Sustainable Chemical Transformations, College of Chemistry, Nankai University, Tianjin 300192, China; zengle@dlut.edu.cn (L.Z.); lhjiang@mail.nankai.edu.cn (L.J.); nkuzmy@mail.nankai.edu.cn (M.Z.)

**Keywords:** triplet-triplet annihilation upconversion, upconversion quantum efficiency, boron-dipyrromethene, Stokes shift, fluorescence quantum yield

## Abstract

Triplet-triplet annihilation upconversion (TTA-UC) has considerable potential for emerging applications in bioimaging, optogenetics, photoredox catalysis, solar energy harvesting, etc. Fluoroboron dipyrrole (Bodipy) dyes are an essential type of annihilator in TTA-UC. However, conventional Bodipy dyes generally have large molar extinction coefficients and small Stokes shifts (<20 nm), subjecting them to severe internal filtration effects at high concentrations, and resulting in low upconversion quantum efficiency of TTA-UC systems using Bodipy dyes as annihilators. In this study, a Bodipy dimer (B-2) with large Stokes shifts was synthesized using the strategy of dimerization of an already reported Bodipy annihilator (B-1). Photophysical characterization and theoretical chemical analysis showed that both B-1 and B-2 can couple with the red light-activated photosensitizer PdTPBP to fulfill TTA-UC; however, the higher fluorescence quantum yield of B-2 resulted in a higher upconversion efficiency (*η*_UC_) for PdTPBP/B-2 (10.7%) than for PdTPBP/B-1 (4.0%). This study proposes a new strategy to expand Bodipy Stokes shifts and improve TTA-UC performance, which can facilitate the application of TTA-UC in photonics and biophotonics.

## 1. Introduction

**Figure 1:**
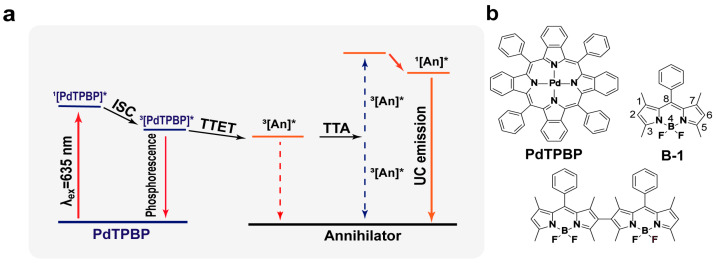
(**a**) Schematic illustration of triplet-triplet annihilation upconversion mechanism; (**b**) Molecular structures of the photosensitizer (PdTPBP) and annihilators (B-1, B-2) in this study.

The photophysical process of photon upconversion converts low-energy photons (long-wavelength light) into high-energy photons (short-wavelength light) [1,2,3,4,5]. Currently, the technology of upconversion luminescence has been extensively utilized in various fields, such as biological imaging [6], 3D printing [7], photocatalysis [8], and solar energy harvesting [9,10]. In particular, triplet-triplet annihilation upconversion (TTA-UC), as the next-generation upconversion material, possesses several distinctive characteristics, such as low excited light power density (~10 mW/cm^2^, sunlight illumination), finely tunable excitation and emission wavelengths, and high upconversion quantum efficiency (*η*_UC_) [11,12,13]. TTA-UC generally consists of two components: photosensitizer (Sen) and annihilator (An). As shown in Figure 1a, in a typical TTA-UC process, the Sen absorbs low-energy incident photons and transits to its singlet excited state (^1^Sen*), and subsequently undergoes an intersystem crossing (ISC) to reach its triplet excited state (^3^Sen*). Then, the energy is transferred from the ^3^Sen* to the annihilator (An) through the triplet-triplet energy transfer (TTET) process. Finally, two triplet excited states of annihilators (^3^An*) collision to operate the triplet-triplet annihilation (TTA) process. One annihilator molecule loses energy and returns to its ground state, while the other annihilator molecule transits to its singlet excited state (^1^An*) and radiates high-energy upconverted photons [14,15].

Developing an efficient TTA-UC system with excellent *η*_UC_ is essential [16,17], in general, which relies on the manipulation of the physiochemical properties of photosensitizers and annihilators [18]. Various types of photosensitizers with intense absorbance and long-lived triplet states, have been developed in prior investigations to significantly improve *η*_UC_ [19,20,21,22]. In addition, adjusting the triplet state (T_1_) of the annihilator can considerably increase the *η*_UC_ via efficiency improvements in the TTET between the photosensitizer and the annihilator. Pioneering studies have been successful in tuning the T_1_ of annihilators for superior *η*_UC_, such as diketopyrrolopyrrole (DPP) and tetracene derivatives [23,24]. However, annihilators are often pure organic dyes with no phosphorescence at even low temperatures, making it difficult to determine their T_1_ state, so it is difficult to systematically regulate the T_1_ energy level of annihilators via a molecular evolution strategy [25]. Boron-dipyrromethene (Bodipy) dyes have been used as model annihilators in TTA-UC because of their high fluorescence quantum yield (Φ*_f_*), robust photostability, and simplicity of chemical functionalization of the molecular structure [26,27,28,29]. For instance, it has been confirmed that the pair of PtTPBP and Bodipy could perform red-to-green or red-to-yellow multicolor photon upconversion [30]. Additionally, perylene and Bodipy moieties were incorporated into the dyad annihilator to widen the Δ*E*_2T1-S1_ and greatly boost *η*_UC_ by driving the TTA process [31]. In addition, the pair of TTA-UC (PtTPBP/Bodipy derivatives) successfully creates a ratio-metric nanothermometer with high thermal sensitivity (7.1% K^−1^) and resolution (0.1 K) that can precisely monitor temperature changes in vivo. This is facilitated by the steric hindrance of the 1,7-dimethyl substituents of Bodipy, which restricts the free rotation of the phenyl moiety at eight sites in Bodipy [32]. However, the small Stokes shift and large molar extinction coefficient of the Bodipy annihilator cause a serious inner-filter effect, which significantly decrease the Φ*_f_* at high concentrations. Furthermore, modulation of the excited state energy level of Bodipy using the π-extension approach could obtain a negative Δ*E*_2T1-S1_ value, such as in 3,5-distyryl Bodipy [26,27,28,29], resulting in a lack of driving force for the TTA process and subsequent decreased upconversion luminescence. The relationship between the T_1_ of the Bodipy annihilator and the size of its π-conjugated molecular surface is so poorly understood that it is difficult to regulate their triplet excited states. Therefore, establishing an effective molecular design strategy to regulate the excited states of Bodipy annihilators, specifically the triplet state, remains challenging but intriguing [33,34].

Herein, we describe a dimerization approach at the 2,6-sites of Bodipy to increase the Stokes shift and enhance the performance of the TTA-UC (Figure 1). Compared to the parent Bodipy (B-1), the S_1_ of B-2 decreased from 2.43 eV to 2.21 eV, while its T_1_ was not reduced significantly after dimerization (1.53 eV vs. 1.52 eV). Calculations based on time-dependent density functional theory (TD-DFT) revealed that the molecular geometry of B-2 tends toward a flat structure in S_1_, resulting in a dramatic decrease in S_1_ energy level due to an extended π-conjugated surface. In contrast, the two Bodipy moieties tend to have an orthogonal structure in T_1_ of B-2, so that the dimerization effect on the T_1_ energy level is negligible. Consequently, the double T_1_ energy levels of the B-2 are considerably greater than its S_1_ (Δ*E*_2T1-S1_ = 0.83 eV), establishing a thermodynamically supported TTA process. Moreover, combining with the red light-absorbing photosensitizer palladium (II) meso-tetraphenyl-tetrabenzoporphyrin (PdTPBP) (Figure 1b), we found that the *η*_UC_ of B-2/PdTPBP (10.7%) was significantly higher than that of B-1/PdTPBP (4.0%).

## 2. Results and Discussion

### 2.1. Characterization of Photophysical Properties of Annihilators

**Scheme 1:**
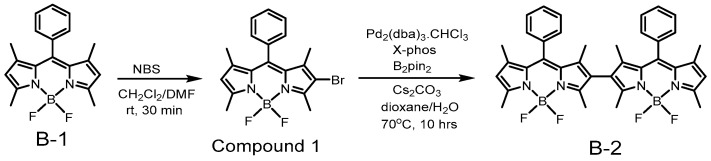
The preparation process of B-2.

B-2 was synthesized by a palladium-catalyzed cross-coupling reaction (Scheme 1) [35]. The reaction yield increased from 14% to 61% when compared to a previously reported Bodipy dimerization catalyzed by FeCl_3_ (supporting information). B-2 is soluble in common organic solvents, such as toluene, DCM, and EtOAc. Nuclear magnetic resonance (NMR), as well as mass spectrometry (MS), were used to identify the molecular structure.

**Figure 2:**
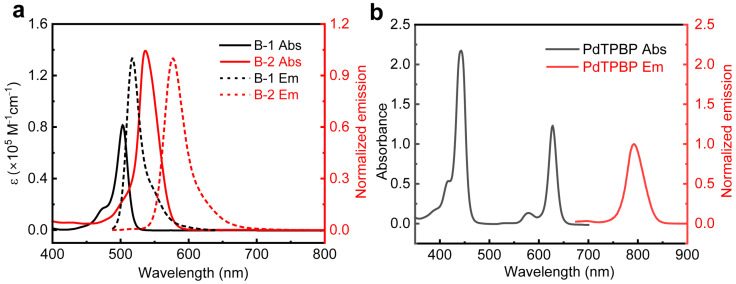
(**a**) UV–vis absorption and fluorescence spectra of B-1 and B-2, λ_ex_ = 470 nm, 10 μM; (**b**) UV–vis absorption and phosphorescence spectra of PdTPBP, λ_ex_ = 635 nm, 10 μM.

As shown in Figure 2a, compared to that of B-1, the UV–vis absorption spectrum of B-2 was red-shifted from 503 to 536 nm, and the molar extinction coefficient was as high as 1.39 × 10^5^ M^−^^1^ cm^−^^1^ (in toluene). The solvent-dependent absorption spectra revealed no change in the absorption profile of B-2, demonstrating that the ground state of B-2 did not undergo charge transfer with the solvent (Figure S1) [36]. The viscosity-dependent absorption spectra of B-1 and B-2 did not exhibit a notable difference (Figure S2).

The fluorescence emission peak of B-2 is 577 nm, which is longer than that of B-1 at 517 nm (Figure 2a). The intersection of the absorption and fluorescence emission spectra shows that the S_1_ of B-1 and B-2 are 2.43 and 2.21 eV, respectively, indicating that the S_1_ of Bodipy was effectively reduced after dimerization (Table 1). The absolute Φ*_f_* of B-1 and B-2 at low and high concentrations were then evaluated. The Φ*_f_* of B-1 was 0.72 at a low concentration (10 μM) but 0.42 at a high concentration (1000 μM). However, B-2 retains its Φ*_f_* even at high concentrations (Φ*_f_* = 0.86, 1000 μM) due to its substantial Stokes shift (B-1 vs. B-2, 539 cm^−1^ vs. 1326 cm^−1^), which suppresses the inner-filter effect at high concentrations. As demonstrated by the polarity-dependent fluorescence emission spectroscopy, the position and width at half maxima of the emission peak of B-1 changed insignificantly, and the fluorescence intensity was not markedly quenched, indicating that intramolecular charge transfer (ICT) and photoinduced electron transfer (PET) had minor effects on the fluorescent properties of B-1 (Figure S3a) [37]. However, the fluorescence emission of B-2 was significantly repressed in acetonitrile, which is presumably due to the low solubility of B-2 in acetonitrile (Figure S3b) [38]. The viscosity-dependent fluorescence spectroscopy of B-2 demonstrated that the fluorescence emission is not viscosity-dependent (Figure S4). To confirm this, we determined the fluorescence lifetime of B-2 at various viscosities (Figure S5). We did not find that the fluorescence lifetime of B-2 got longer as the viscosity went up. This means that the rotation between the two Bodipy moieties or the 8-site phenyl substitutes does not cause excited-state cone crossings, which would quench fluorescence emission [39].

In addition, we further investigated the redox properties of B-1 and B-2 by cyclic voltammetry versus the Ag/Ag^+^ electrode. The oxidation/reduction potentials of B-1 and B-2 were +0.90/−1.59 V and +0.84/−1.51 V, respectively (Figure S6), demonstrating that Bodipy dimerization does not change the redox potential of the annihilator and, thus, does not result in intramolecular charge separation, which causes fluorescence quenching [40].

Next, the triplet-excited state of B-2 was investigated. Due to the high Φ*_f_* and extremely low triplet state quantum yield of B-2, we were unable to directly observe its phosphorescence emission to determine the T_1_ energy level. The T_1_ of B-2 was approximated using the triplet sensitization bracketing technique. PdTPBP (T_1_ = 1.55 eV) and PtTNP (T_1_ = 1.36 eV) are two metalloporphyrins with high phosphorescence quantum yield that have been chosen as triplet energy donors. In the presence of 1000 µM B-1 or B-2, we measured the steady and transient photoluminescence spectra of the triplet energy donor. In the presence of B-2, the phosphorescence of PdTPBP at 800 nm decreased by 50.9% (Figure S7), whereas the phosphorescence intensity of PtTNP remained unchanged at 913 nm (Figure S8a). In addition, the phosphorescence intensity of PdTPBP decreased by 33.1% in the presence of B-1, while that of PtTNP was unaffected. The aforementioned experimental results indicate that the T_1_ state energy levels of B-1 and B-2 are between 1.36 eV and 1.55 eV.

**Figure 3:**
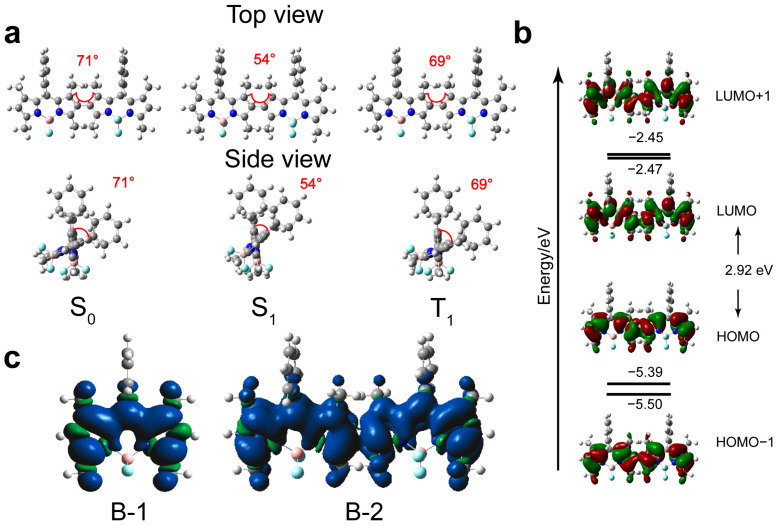
Theoretical chemical calculation results. (**a**) Calculated S_0_, S_1_, and T_1_ configurations of B-2 in toluene, highlighting the dihedral angles between two Bodipy moieties, top panel is the top view, bottom panel is the side view. (**b**) Selected frontier molecular orbitals of B-2 including HOMO, LUMO, HOMO−1, and LUMO+1. (**c**) Triplet state spin density surfaces of B-1 and B-2, respectively, in toluene at the optimized triplet state molecular geometric configurations. Calculated with Gaussian 09 based on the DFT-B3LYP/6-31G.level.

To gain a deeper understanding of the excited state of B-2, we calculated its properties using time-dependent density functional theory (TD-DFT). Optimization of the ground state (S_0_) configuration of B-2 revealed that the dihedral angle between the two Bodipy moieties is 71° (Figure 3a), suggesting that the steric hindrance of the four methyl substituents in B-2 prevents free rotation between the two Bodipy moieties. The scanning of potential energy surface confirms that the energy of B-2 rises rapidly with the increased co-planarization degree between the two Bodipy moieties (Figure S9). The vertical excitation energy of B-2 corresponds well to its UV–vis absorption spectrum (Table S1), showing that the theoretical model used in the DFT-calculations is valid [41]. In contrast to the optimal conformation of the S_0_, the S_1_ conformation of B-2 discloses that the dihedral angle between the two Bodipy moieties tends to decrease from 71° to 54° (Figure 3a), indicating that the red-shift of the fluorescence emission of B-2 is associated to the extensive π-conjugated surface between the two Bodipy moieties. Furthermore, the significant differences between S_1_ and S_0_ in the geometrical configuration result in a large Stokes shift of B-2 [42]. Both Bodipy moieties of B-2 have a frontier orbital electron density population, as calculated by the HOMO and LUMO orbitals of B-2 (Figure 3b). This conclusion is attributable to the enhancement of the π-conjugated surface between the Bodipy moieties resulting from the lowering of the dihedral angle, which is directly associated with the prolonged fluorescence emission wavelength of B-2 [43]. The triplet state of B-2 is then investigated using TD-DFT. As shown in Figure 3a, the dihedral angle between the two Bodipy moieties is 69° in the optimized T_1_ conformation of B-2, which is no change in the molecular geometry configuration as compared to S_0_. Calculation of the triplet state energy levels of B-2 shows that its T_1_ and T_2_ states are 1.52 eV and 1.53 eV, respectively. The T_1_ and T_2_ energy levels are close together due to a lack of π-conjugated electron delocalization between the two Bodipy moieties of B-2, resulting in a degenerate T_1_ state [42]. The triplet spin density of B-2 is calculated to be populated in both Bodipy moieties, which further confirmed the abovementioned results (Figure 3c) [41]. In this way, B-1 and B-2 exhibit similar T_1_ state energy levels (Table S1). The results of the preceding experimental experiments are consistent with the outcomes of the theoretical chemical calculations.

In the case of Bodipy dyes, both S_1_ and T_1_ decrease as the π-conjugation surface increases, but T_1_ decreases more significantly [26,27,28,29]. This led to a negative Δ*E*_2T1-S1_ value for most Bodipy derivatives, limiting their utilization in TTA-UC [15]. Despite the fact that a dyad annihilator composed of perylene and Bodipy was developed to address this issue, intramolecular charge transfer causes fluorescence quenching and, as a result, reduced TTA-UC efficiency in highly polar solvents [43]. By regulating the molecular geometric configuration of S_1_ and T_1_, our proposed Bodipy dimerization strategy raises the Stokes shift of the annihilator to prevent dose-mediated upconversion quenching [24].

### 2.2. TTA-UC Properties of Annihilators

Following that, we chose the red light-absorbing PdTPBP (T_1_ energy level = 1.55 eV [44]) as the photosensitizer to investigate the TTA-UC in relation to B-2. Since PdTPBP has a long triplet state lifetime, its own TTA is not conducive to TTET with the annihilator; thus, a low dose of PdTPBP (10 μM) is used. Because of the close dependence of the TTET and TTA processes on the annihilator concentration, we first optimized the B-2 concentration in TTA-UC (Figure S10) [17]. When the concentration of B-2 exceeded 1000 μM, the TTA-UC intensity stopped increasing and even decreased in the concentration interval 1000–1500 μM. This might be because of the self-absorption and fluorescence quenching with the high dose of B-2 [24]. As a result, we measured the *η*_UC_ of B-1 and B-2 at 1000 μM, which were determined to be 10.7% and 4.0%, respectively (excited by a 635 nm laser, power intensity = 1267.5 mW/cm^2^).

**Figure 4:**
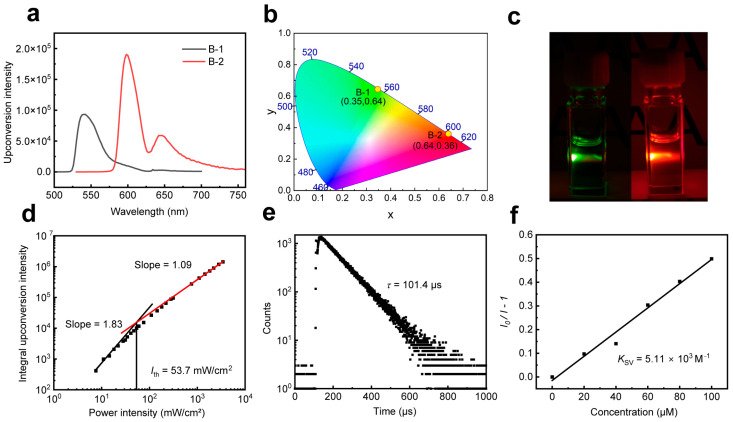
Upconversion properties of the annihilators. (**a**) Upconversion emission spectra of B-1 and B-2 in toluene, *λ*_ex_ = 635 nm (1267.5 mW/cm^2^); (**b**) CIE diagram showing the adjustable upconversion emission colors; (**c**) Upconversion pictures of B-1 and B-2 with PdTPBP; (**d**) Power-dependence of TTA-UC for PdTPBP/B-2, a slope of 1.83 (black, quadratic) and a slope of 1.09 (red, linear), *I*_th_ is 53.7 mW/cm^2^; (**e**) Upconversion lifetime decay trace of PdTPBP/B-2 at 600 nm, in deaerated toluene, PdTPBP (10 µM); (**f**) Stern-Volmer plots of PdTPBP in response to B-2 addition in toluene.

As demonstrated in Figure 4a, the TTA-UC peak of B-2 was redshifted to 600 nm compared to B-1. In particular, the TTA-UC spectrum of B-2 exhibits significantly lower intensity at 630 nm compared to the fluorescence spectrum of B-2. This is due to the strong absorption of PdTPBP at this position (Figure S11), indicating the “emission–reabsorption” effect and that the TTA-UC efficiency of PdTPBP/B-2 should be greater than 10.7%. We further evaluated the color of TTA-UC using Commission Internationale de l’Eclairage (CIE) coordinates (Figure 4b). The CIE coordinates of B-1 and B-2 are (0.35, 0.64) and (0.64, 0.36), respectively, indicating that the molecular structure of the annihilator can be finely tuned to produce multicolor TTA-UC. Simultaneously, we observed the TTA-UC colors of B-1 and B-2 in green and yellow, respectively, with the naked eye under low-power red illumination (Figure 4c). The threshold power intensity (*I*_th_) is a critical TTA-UC parameter. The integrated TTA-UC intensity (*I*_UC_) has a linear relationship with the incident light power intensity (*I*_ex_) when *I*_ex_ is greater than *I*_th_ and a nonlinear quadratic relationship when *I*_ex_ is less than *I*_th_. The power-dependent TTA-UC spectra of PdTPBP/B-2 are shown in Figure S12, with a significant increase in *I*_UC_ with increasing *I*_ex_. We found a quadratic relationship between the *I*_UC_ and the *I*_ex_ in the low-power intensity region by logarithmic plotting (Figure 4d). This further demonstrates that the B-2 acts as an annihilator to achieve the red-to-yellow TTA-UC. The *I*_th_ of B-2 (53.7 mW cm^−2^) is lower than B-1 (76.9 mW cm^−2^), suggesting that expanding the Δ*E*_2T1-S1_ contributes to the development of TTA-UC pairs with lower *I*_th_, which are desirable for solar energy harvesting, photoredox catalysis, and upconversion bioimaging [6]. In addition, the TTA-UC delayed fluorescence lifetime (*τ*_DF_) of B-1 and B-2 were measured. The *τ*_DF_ of B-1 and B-2 were 445.5 μs and 101.4 μs, respectively, which were three orders of magnitude longer than their own *τ_f_*, confirming that this long-lived luminescence was derived from the TTA-UC process [45].

We further measured the TTET efficiency (Φ_TTET_) of PdTPBP (10 µM) with B-1 (1000 µM) or B-2 (1000 µM) to gain a better understanding of the TTA-UC procedure. The Φ_TTET_ of PdTPBP/B-2 (66.9%) is higher than that of PdTPBP/B-1 (49.1%) (Table 2). In addition, we calculated the triplet state molecular collision cross-section areas of B-1 and B-2 based on DFT theory [46]. The triplet state collision cross-sections of B-1 and B-2 are 1205.0 Bohr^2^ and 2206.1 Bohr^2^, respectively. The calculation results show that although B-2 has T_1_ energy levels similar to B-1, the collision surface of B-2 is greatly extended, which facilitates the Dexter-type triplet energy transfer. Therefore, there is a higher TTET efficiency between PdTPBP and B-2. To further validate our hypothesis, we measured the Stern–Volmer quenching constants (*k*_sv_) of B-1 and B-2 for PdTPBP phosphorescence and then calculated their bimolecular quenching constants (*k*_q_). As shown in Table 2, the *k*_sv_ of B-2 is 9.0 times higher than those of B-1, and the *k*_q_ is as high as 2.1 × 10^7^ M^−1^ s^−1^. The large *k*_sv_ of B-2 suggests that the dimerization strategy of Bodipy can promote TTET between photosensitizer and annihilator.

Finally, we measured the normalized triplet annihilation efficiencies (*η*_TTA_) of B-1 and B-2 using a well-established protocol [47]. The *η*_TTA_ of PdTPBP/B-1 and PdTPBP/B-2 are 88.0% (39,570 mW/cm^2^) and 82.0% (39,570 mW/cm^2^), respectively (Figure S13).

## 3. Materials and Methods

### 3.1. Preparation Process for B-2 [35]

A 25 mL three-necked flask was filled with 2-bromo-Bodipy (compound **1**) (16.2 mg, 0.05 mmol), X-phos (19.0 mg, 40 mol), bis(pinacolato) diboron (B_2_pin_2_) (5.1 mg, 0.02 mmol), Cs_2_CO_3_ (65 mg) 1,4-dioxane (5 mL), and H_2_O (200 µL). Then, the mixture was degassed with argon for 10 min, followed by the addition of Pd_2_(dba)_3_·CHCl_3_ (5.2 mg, 5.0 µmol) and another argon degassing for 5 min. After 10 h of reaction at 70 °C, the solvent was evaporated, and the residue was purified using column chromatography on silica with V_hexane_/V_DCM_ = 1:1 to yield 9.5 mg (yield: 61%). ^1^H NMR (400 MHz, CDCl_3_) (ppm): 7.53–7.43 (m, 6H), 7.34–7.22 (m, 4H), 5.99 (s, 2H), 2.56 (s, 6H), 2.35 (s, 6H), 1.37 (s, 6H), 1.12 (s, 6H). ^13^C NMR (100 MHz, CDCl_3_) (ppm): 156.06, 154.71, 143.67, 141.74, 141.25, 135.09, 131.80, 131.28, 129.31, 129.18, 129.07, 128.01, 127.89, 124.78, 121.52, 14.70, 14.44, 13.37, 12.90; MS (MALDI) for B-2 (C_38_H_36_B_2_F_4_N_4_) m/z = 646.31 (calculated), 646.31 (observed).

### 3.2. Measurement of the Fluorescence Quantum Yields (Φ_f_) of B-1 and B-2

The literature has previously reported the fluorescence quantum yields of B-1 and B-2 using the relative method with fluorescein as the reference [48]. We utilized the absolute method based on the integrating sphere to determine the fluorescence quantum yields of B-1 and B-2 with greater precision. The absolute method was used to measure the Φ*_f_* in an FLS1000 photoluminescence spectrometer with an integrating sphere and a xenon lamp as the light source. Φ*_f_* is the total number of emitted photons divided by the total number of absorbed photons. We determined the Φ*_f_* of the annihilators at concentration of 10 μM, 1000 μM as well as the annihilators (1000 μM) in the presence of PdTPBP (10 μM). Since the fluorescence emission spectra of B-2 overlaps with the ultraviolet–visible (UV–vis) absorption spectrum of PdTPBP, the Φ*_f_* of the mixture solution B-2/PdTPBP is lower than that of B-2 alone.

### 3.3. Measurement of Upconversion Efficiency (η_UC_)

The *η*_UC_ was calculated with the reference Ru(bpy)_3_Cl_2_·6H_2_O, which has a photoluminescence quantum yield (Φ*_p_*) of 0.028 in water [49]. The TTA-UC pairs and Ru(bpy)_3_Cl_2_·6H_2_O were excited using a 635 nm diode laser (39.8 mW) and a 450 nm diode laser (39.8 mW), respectively. We have uniformed the sensitivity of the fluorometer at different wavelengths. The *η*_UC_ was calculated using the following equation: eq 1, where *η*_UC_, Φ*_f_*, *A*_std_, *A*_sam_, *I*_std_, *I*_sam_, *η*_std_, and *η*_sam_ represent the upconversion efficiency, fluorescence quantum yield of reference, reference absorbance (450 nm), PdTPBP absorbance (635 nm), reference integrated photoluminescence intensity, the integral area of the upconversion spectrum, and the refractive index of H_2_O (1.333). Note that the theoretical maximum of *η*_UC_ is standardized to be 1 (100%).

(1) $$\eta_{\mathrm{UC}}=2\;\times \;\Phi f_{\;}\times \;\frac{A_{std}}{A_{sam}}\times \frac{I_{sam}}{I_{std}}\times {\left(\frac{\eta_{sam}}{\eta_{std}}\right)}^{2}$$

### 3.4. Theoretical Chemical Calculation [50]

All theoretical calculations were performed using the Gaussian 09 program. The ground state geometries of the B-1 and B-2 were optimized using density functional theory (DFT) based on B3LYP/6-31G(d) level. Based on the optimized ground state geometry, the energies of the lowest singlet and triplet excited states were calculated using the TD-DFT method.

## 4. Conclusions

In conclusion, the new annihilator B-2 was synthesized using the Bodipy dimerization strategy at the 2,6 sites. The *η*_UC_ was elevated from 4.0% to 10.7% in comparison to the conventional B-1 annihilator. Through theoretical chemical calculations and spectroscopic characterization, it was confirmed that B-2 not only increases the fluorescence quantum yield but also increases the triplet collisional surface to improve the Φ_TTET_. These above features are extremely important for enhancing the *η*_UC_. Thus, this research provides not only a new molecular design strategy for finely regulating the excited state properties of Bodipy-based annihilators but also a new approach for the development of efficient TTA-UC, which will surely promote the use of TTA-UC in photonics and biophotonics fields.

## Figures and Tables

**Table 1:** The photophysical parameters of B-1 and B-2 in toluene.

Compound	*λ*_abs_ ^a^ (nm)	*ε* ^b^	*λ*_em_^c^ (nm)	Φ*_f1_* ^d^ (%)	Φ*_f2_* ^e^ (%)	*τ_f_* ^f^(ns)	S_1_^g^ (eV)	Δ*E*_2T1-S1_ ^h^ (eV)
B-1	503	0.81	517	72	42	3.52	2.43	0.63
B-2	536	1.39	577	92	86	3.25	2.21	0.83

^a^ absorption peak; ^b^ molar extinction coefficient, 10^5^ M^−1^ cm^−1^; ^c^ fluorescence emission peak; ^d^ fluorescence quantum yield, 10 μM; ^e^ fluorescence quantum yield, 1000 μM; ^f^ fluorescence lifetime; ^g^ single excited energy level was determined as the crossing point of the absorption and fluorescence emission spectra; ^h^ thermodynamic driving force for TTA, T_1_ state energy level was calculated with TD-DFT.

**Table 2:** The TTA-UC parameters of PdTPBP/B-1 and PdTPBP/B-2 in deaerated toluene.

Compound	*η*_UC_ ^a^	Φ_TTET_ ^b^ (%)	*η*_TTA_ ^c^ (%)	Φ*_f_* ^d^ (%)	*I*_th_ ^e^	*k*_sv_ ^f^	*k*_q_ ^g^	*τ*_DF_ ^h^ (µs)	T_1_^i^ (eV)
B-1	4.0	49.1	88.0	34.3	76.9	0.57	0.23	445.5	1.53
B-2	10.7	66.9	82.0	60.3	53.7	5.11	2.10	101.4	1.52

^a^ TTA-UC efficiency at 635 nm CW excitation; ^b^ triplet-triplet energy transfer efficiency; ^c^ normalized triplet-triplet annihilation efficiency, 39,570 mW/cm^2^; ^d^ absolute fluorescence quantum yields of annihilators (1000 µM) in the presence of PdTPBP (10 µM); ^e^ threshold power intensity, mW/cm^2^; ^f^ Stern–Volmer quenching constant, in 10^3^ M^−1^; ^g^ bimolecular quenching constants, in 10^7^ M^−1^ s^−1^; ^h^ upconversion fluorescence lifetime. ^i^ T_1_ state energy levels were calculated with TD-DFT.

## Data Availability

The data reported are contained within the manuscript and Supplementary Materials.

## Supplementary Materials

The following supporting information can be downloaded at https://www.mdpi.com/article/10.3390/molecules28145474/s1, Figure S1: absorption spectra of annihilators in different solvents; Figure S2: absorption spectra of annihilators in solutions of different viscosities; Figure S3: Fluorescence spectra of annihilators different solvents; Figure S4: Fluorescence spectra of annihilators with different viscosities; Figure S5: Fluorescence lifetime of annihilators with different viscosities; Figure S6: Cyclic voltammograms of B-1 and B-2; Figure S7: Phosphorescence emission spectra of PdTPBP without annihilators and in the presence of annihilators (determination of TTET efficiency); Figure S8: Phosphorescence emission spectra and phosphorescence lifetime of PtTNP without annihilators and in the presence of annihilators; Figure S9: Ground state potential energy curves of B-2, as a function of the dihedral angle between two moieties B-2 (step: 10°); Figure S10: (a) The upconversion emission spectra of PdTPBP (10 µM) and different concentrations of B-1 in degassed toluene, (b) quantitative analysis of the relationship between upconversion intensity and concentration of B-1. *λ*ex = 635 nm, (c) The upconversion emission spectra of PdTPBP (10 µM) and different concentrations of B-2 in degassed toluene, (d) quantitative analysis of the relationship between upconversion intensity and concentration of B-2. *λ*ex = 635 nm; Figure S11: Fluorescence spectra of B-2 (1 mM) with or without photosensitizer; Figure S12: (a) Incident light power dependence study of TTA-upconversion analysis; Figure S13: ηTTA determination of annihilator compounds; Figure S14: The excitation spectrum of B-2 in toluene, *λ*em = 600 nm; Figure S15: 1H-NMR (400 MHz, CDCl_3_) of B-1; Figure S16; ^1^H-NMR (400 MHz, CDCl_3_) of B-2; Figure S17: ^13^C-NMR (100 MHz, CDCl_3_) of B-2; Figure S18: HRMS (MALDI) of B-2; Table S1: Density functional theory (DFT) calculation for the annihilators. Table S2: S1 and T1 energy levels of B-1, B-2, obtained by experiments, CAM-B3LYP, and B3LYP calculations. The reference [51,52,53,54,55,56,57] is in the Supplementary Material.
