# Supplementary material for: Bodipy Dimer for Enhancing Triplet-Triplet Annihilation Upconversion Performance
Source: Molecules. 2023 Jul 18;28(14):5474. doi: 10.3390/molecules28145474 (PMC10384713; doi:10.3390/molecules28145474)
Supplement: Supplementary file 1 [file molecules-28-05474-s001.zip › molecules-2488004-SI.pdf]

Supporting information for:

Bodipy dimer for enhancing triplet-triplet an-nihilation upconversion performance

Min Gao[1,2], Le Zeng[2], Lin-Han Jiang[2], Ming-Yu Zhang[2], Yong Chen[*1] and Ling Huang[*2]

[1] Jiangxi Key Laboratory for Microscale interdisciplinary Study, Institute for Advanced Study, Nanchang University, Nanchang, Jiangxi, 330031, China

[2] Research Center for Analytical Sciences and Tianjin Key Laboratory of Biosensing and Molecular Recognition, College of Chemistry, Nankai University; Haihe Laboratory of Sustainable Chemical Transformations, Tianjin 300192, China

Chemicals and Characterization.

Unless otherwise stated, reagents used in the synthesis were commercially available and used as received. **B-1** was synthesized according to the literature.[1] PdTPBP was purchased from Frontier Scientific. 2,4-dimethylpyrrole was purchased from HEOWNS. Benzoyl chloride was purchased from *J&K* Scientific. Trifluoroacetic acid was purchased from MACKLIN. Boron trifluoride diethyl etherate ($BF_3.Et_2O$) and tetrabutylammonium hexafluorophosphate were purchased from TCI chemicals. Triethylamine (TEA) was purchased from AMAR CHEMICAL. Anhydrous $FeCl_3$ was purchased from RHAWN. Deuterated chloroform ($CDCl_3$) was purchased from Innochem. Toluene was purchased from Damao chemical reagent factory. Ferrocene and methyl cyanide (MeCN) were purchased from Tianjin Medema Biotechnology Co., Ltd. Ethyl acetate (EtOAc), ethanol (EtOH), dichloromethane (DCM), hexane, and silica gel (40–63 μm) were purchased from Tianjin Hedong District Guangda Service Department.

^1H and ^{13}C NMR spectra were recorded with Bruker AVANCE Ⅲ 400 MHz spectrometer ($CDCl_3$ as solvents, TMS as a standard, δ = 0.00 ppm). The high-resolution mass spectrum (HRMS) was measured on a Bruker Autoflex III TOF/TOF200 MALDI-TOF. UV-vis absorption spectra were recorded on a Shimadzu UV-3600 Plus spectrophotometer. Steady-state fluorescence spectra were measured on an Agilent Technologies Cary Eclipse fluorescence spectrometer. Triplet-triplet annihilation upconversion spectroscopy,

fluorescence quantum yield, and upconversion lifetime were measured on an Edinburgh FSL1000 photoluminescence spectrometer, 635 nm diode laser (CNI, MDL-D-635-2W) as excitation light source, the determination of upconversion efficiencies also required a 450 nm diode laser (CNI, MDL-III-450-1W). Cyclic voltammograms (CV) and differential pulse voltammograms (DPV) were measured on LK5100 electrochemical analysis system (working electrode: glassy carbon, reference electrode: Ag/$AgON_3$, counter electrode: platinum wire, scan rates were 100mV/s under argon atmosphere).

Preparation of B-1[1].

2, 4-Dimethylpyrrole (2.09 g, 22 mmol) and benzoyl chloride (1.41 g, 10 mmol) were added to a 500 mL round bottom flask containing 300 mL of argon degassed anhydrous dichloromethane (DCM). The mixture was stirred at room temperature for 8 h. Add 12 mL of anhydrous triethylamine (TEA) dropwise at 0°C, stir for 30 min, and then add $BF_3.OEt_2$ (12 mL) dropwise at 0°C, return to room temperature. The mixture was stirred continuously for 2 h. Half of the solvent was removed and saturated aqueous sodium bicarbonate solution was added and stirred overnight. The reaction mixture was washed with water and extracted with DCM. The organic phase was dried with Na_2SO_4. The solvent was evaporated and the residue was purified by silica gel column chromatography (PE/DCM = 1:1 v/v) to obtain an orange solid of 1.10 g (34%). ^1H NMR (400 MHz, $CDCl_3$) δ (ppm): 7.52-7.43 (m, 3H), 7.31-7.23 (m,2H), 5.98 (s, 2H), 2.56 (s, 6H), 1.37 (s, 6H).

Preparation of 1[2]**.**

To a solution of 1 (97 mg, 0.30 mmol) in DMF/CH_2Cl_2 (15 ml/15 ml) was added NBS (64 mg, 0.36 mmol). The reaction mixture was stirred at room temperature for 30 min. The reaction mixture was washed with brine and then extracted with CH_2Cl_2. The organic phase was dried over Na_2SO_4. The solvent was evaporated and the residue was purified by column chromatography on silica with (hexane/ CH_2Cl_2 = 1:2 v/v) to obtain an orange solid.

Preparation of B-2 with $FeCl_3$ [3].

To a solution of B-1 (100 mg, 0.31 mmol) in dry CH_2Cl_2 (20 ml) was added anhydrous $FeCl_3$ (140 mg, 0.86mmol). The orange solution rapidly turns deep green-red-mauve. The reaction mixture was stirred at room temperature for 15min. Methanol was added and stirred for 10 min to quench the reaction. The reaction mixture was washed with water and then extracted with CH_2Cl_2. The organic phase was dried over Na_2SO_4. The solvent was evaporated and the residue was purified by column chromatography on silica with (hexane/ CH_2Cl_2 = 1:2 v/v) to obtain a mauve solid 14 mg (14 %).

Calculation of the standard oxidation/reduction potential of the annihilators [4].

Standard oxidation (E_{ox})/reduction (E_{red}) potentials were obtained from the cyclic voltammetry (CV) as the sum of half of the anodic (E_{pa}) and cathodic (E_{pc}) peak potentials, as shown below.

$E_{ox} = (E_{pa} + E_{pc}) / 2$……………………………………………………………......(1)

$E_{red} = (E_{pa} + E_{pc}) / 2$……………………………………………………..……(2)

The Stern-Volmer quenching plot experiment

The k_{sv} constants were calculated by eq 3 [5], where I_0 and I_t represent the phosphorescence intensity of PdTPBP in the absence of annihilators and the phosphorescence intensity of PdTPBP in the presence of annihilators. Q is the concentration of annihilators. Bimolecular quenching constants (k_q) were calculated by eq 4.[11] The τ_t is the phosphorescence lifetime of PdTPBP in deoxygenated toluene. The triplet excited state lifetime of PdTPBP is 243.5 μs. Using xenon lamp as excitation light source (635 nm).

$$\frac{I_0}{I_t} = 1 + k_{sv}\,Q \quad (3)$$

$$k_{sv} = k_q \times \tau_t \quad (4)$$

Triplet-triplet energy transfer (TTET) efficiency measurements.

The PdTPBP-to-annihilators triplet-triplet energy transfer quantum efficiency (Φ_{TTET}) was calculated from the measurements of the PdTPBP residual phosphorescence in the absence of the annihilators (I_0) and in the presence of annihilators (I_t, 1 mM), using Xenon lamp as excitation light source (635 nm), calculated with following eq 5[6, 7].

$$\Phi_{TTET} = 1 - \frac{I_t}{I_0} \quad (5)$$

The triplet lifetimes of the annihilators and normalized triplet-triplet annihilation efficiency (η_{TTA}) measurements [8, 9].

The triplet lifetimes of the annihilators and η_{TTA} were determined using time-resolved emission measurements. To account for the first- and second-order decays, equation 6 was used for fitting the data.

$$I(t) \propto [^3A^*(t)]^2 = ([^3A^*]_0 \frac{1-\beta}{exp_{(t/\tau_T)}-\beta})^2 \quad (6)$$

I(t) is the time-dependent upconversion emission intensity, $[^3A^*]$ is the initial annihilator triplet concentration, t is time, β is a dimensionless parameter between 0 and 1, indicating the part of the initial decay dominated by second-order channels, as defined by eq 7, β represents a system's η_{TTA} (with a possible maximum of 100%), τ_T is the annihilator triplet lifetime, The analytical expression for β is given by equation 7.

$$\beta = \frac{2k_{TTA}[\,^3A^*]_0}{k_T+2k_{TTA}[\,^3A^*]_0} \quad (7)$$

Here, k_T (=1/τ_T) is the intrinsic first-order rate constant of annihilator triplet decay, $[^3A^*]_0$ is the initial annihilator triplet concentration, k_{TTA} is the rate constants for the second-order TTA decay. In the case of annihilators, upconversion emission is measured, which is the result of the second-order TTA events. The initial decay through the second-order channel is partially dependent on the excitation intensity, as this controls the initial concentration of the excited triplets. Thus, emission measurements are performed at several different intensities with the 635 nm pulsed laser, and the kinetics are then fitted globally with equation 4 using a shared τ_T, but β is fitted to each trajectory individually.

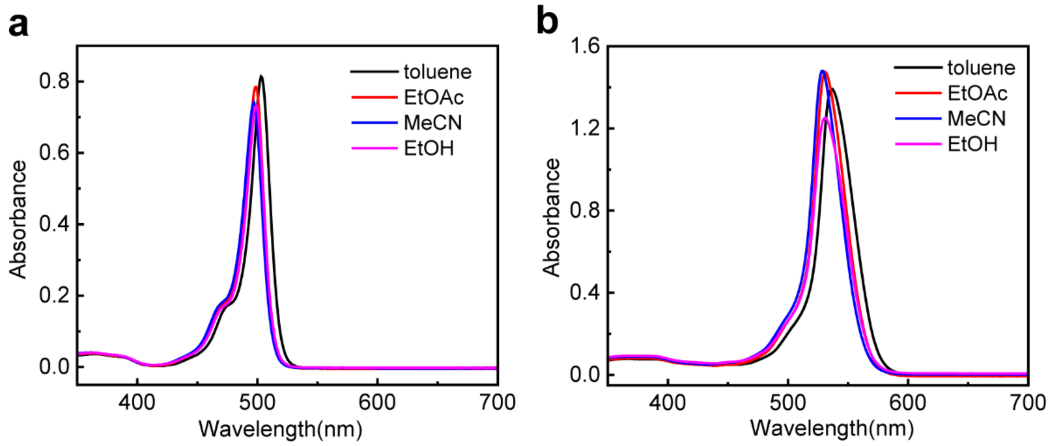

Figure S1. UV-Vis absorption spectra of annihilators in toluene, ethyl acetate, acetonitrile, and ethanol, respectively, 10 μM, (a) B-1, (b) B-2.

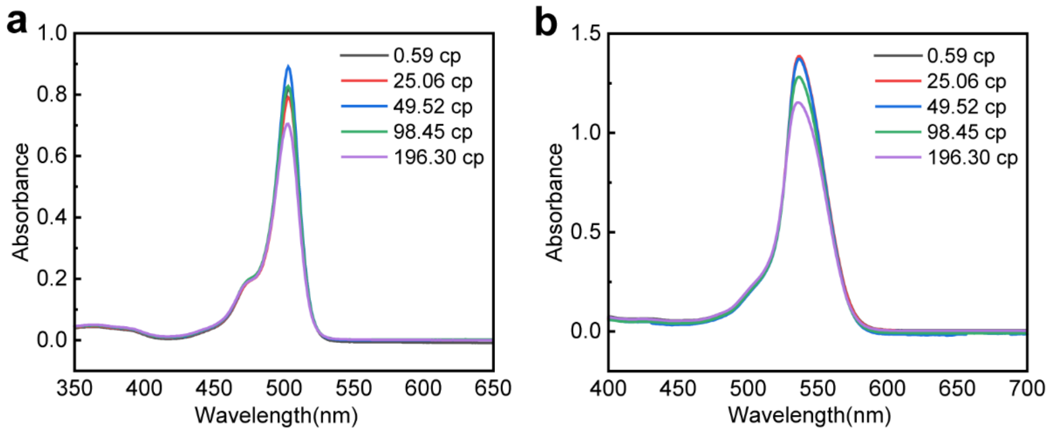

Figure S2. UV-Vis absorption spectra of annihilators in solutions of different viscosities, 10 μM. (a) B-1, (b) B-2.

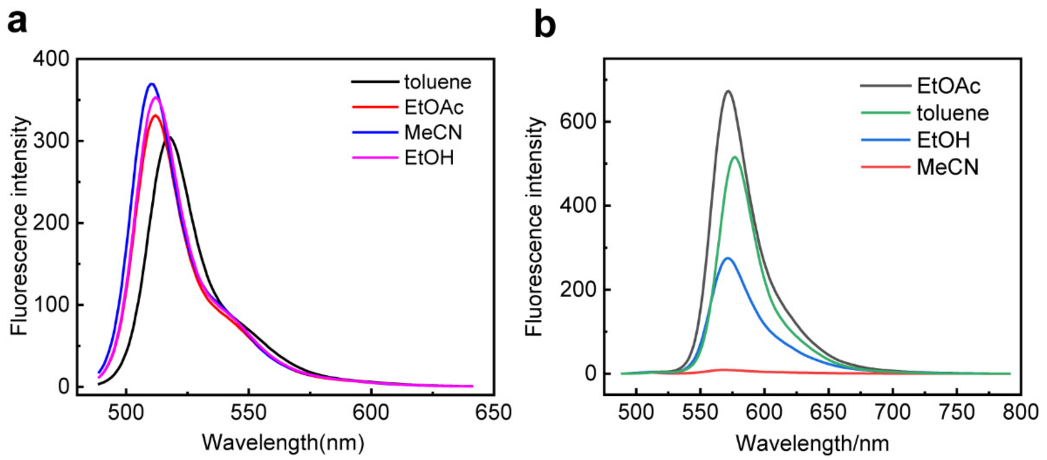

Figure S3. Fluorescence spectra of annihilators in toluene, ethyl acetate, acetonitrile, and ethanol, respectively, 10 μM, λ_{ex} = 470 nm, (a) B-1, (b) B-2.

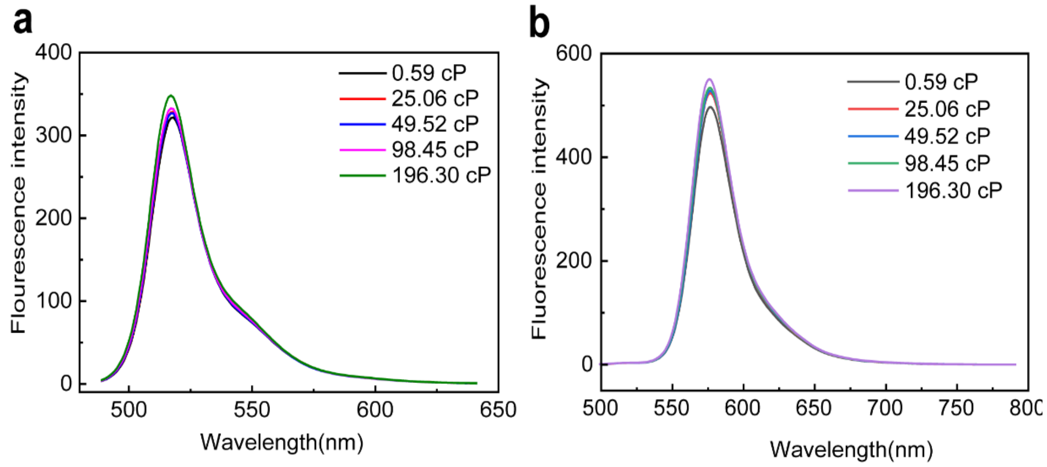

Figure S4. Fluorescence spectra of annihilators with different viscosities, 10 μM, λ_{ex} = 470 nm, (a) B-1, (b) B-2.

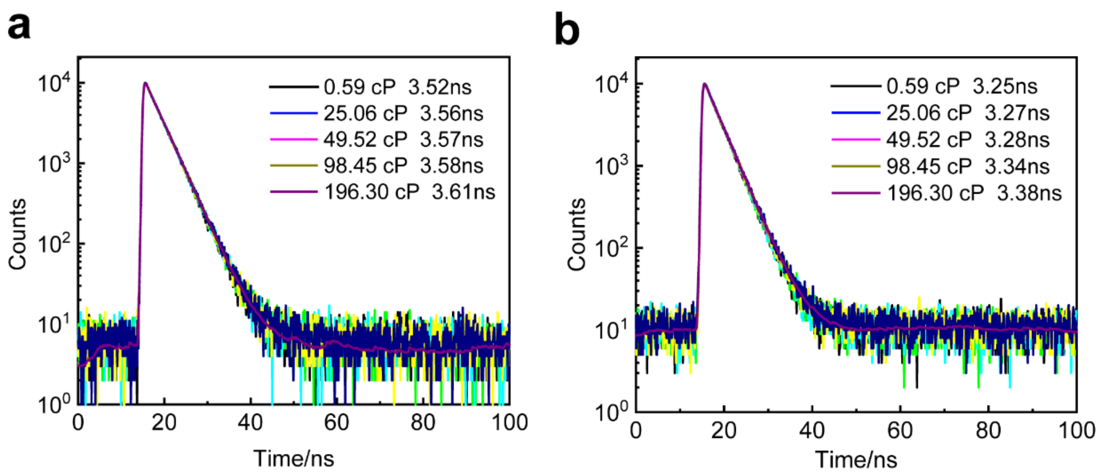

Figure 5. Fluorescence lifetime of annihilators with different viscosities, 10 μM, λ_{ex} = 470 nm, (a) B-1, (b) B-2.

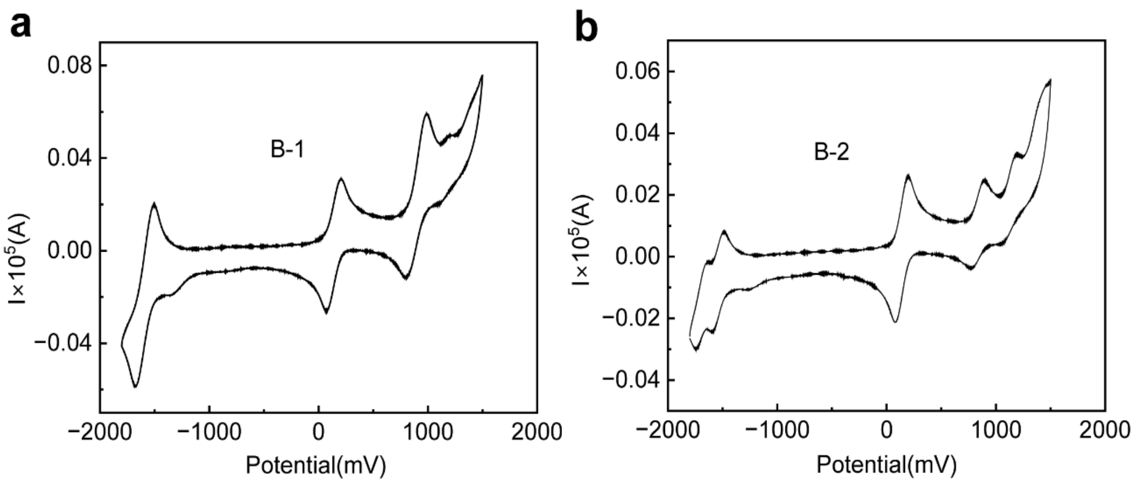

Figure S6. Cyclic voltammograms of 2 mM B-1 and 1 mM B-2, ferrocene as internal standard, supporting electrolyte is 0.1 M Bu_4NPF_6, the solvent is deoxygenated CH_2Cl_2, scan rate is 100mV/s. (Working electrode: glassy carbon electrode; reference electrode: Ag/AgN0_3; counter electrode: platinum wire electrode, under argon atmosphere.)

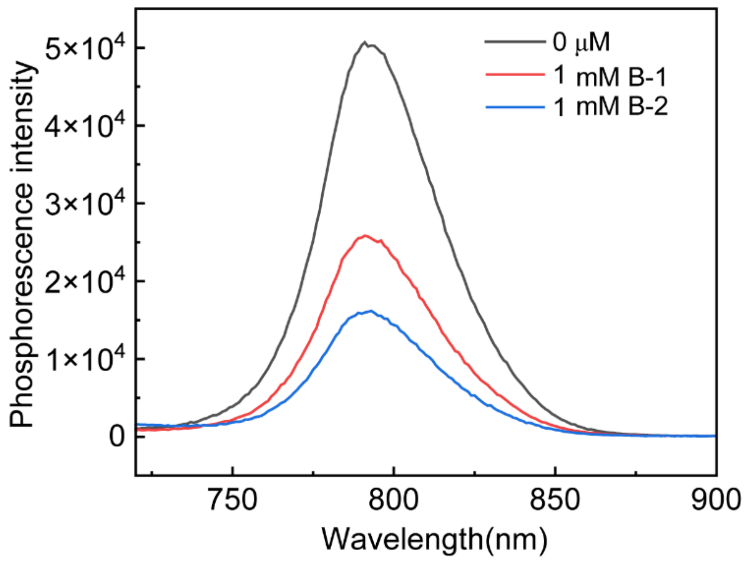

Figure S7. Phosphorescence emission spectra of PdTPBP without annihilators and in the presence of annihilators (determination of TTET efficiency), λ_{ex} = 635 nm, c(PdTPBP) = 10 μM, in degassed toluene.

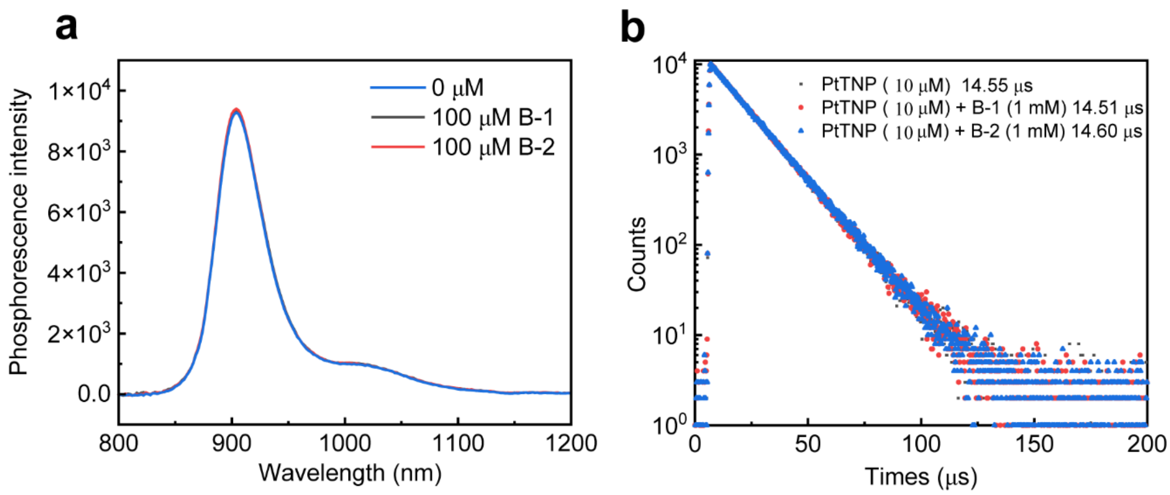

Figure S8. (a) Phosphorescence emission spectra of PtTNP without annihilators and in the presence of annihilators, λ_{ex} = 635 nm, c (PtTNP) = 10 μM, in degassed toluene; (b) Phosphorescence lifetime variation of PtTNP with different annihilator concentrations, in degassed toluene, λ_{ex} = 635 nm.

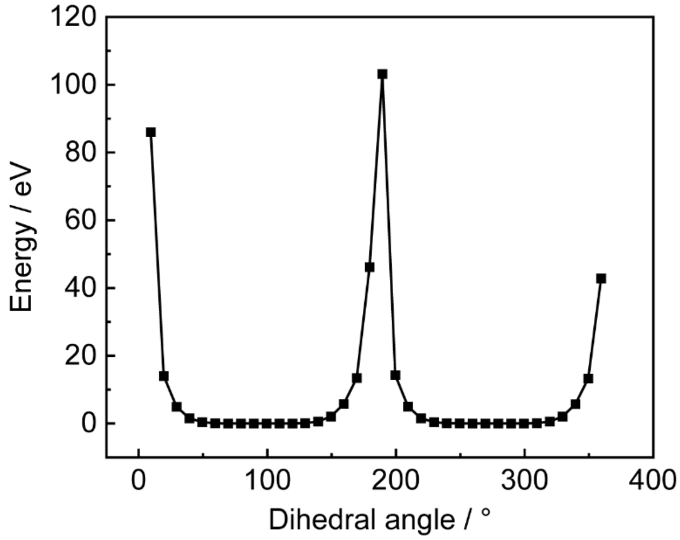

Figure S9. Ground state potential energy curves of B-2, as a function of the dihedral angle between two moieties B-2 (step: 10°), predicted from density functional theory (DFT) based on B3LYP/6-31G(d) level in toluene with Gaussian 09.

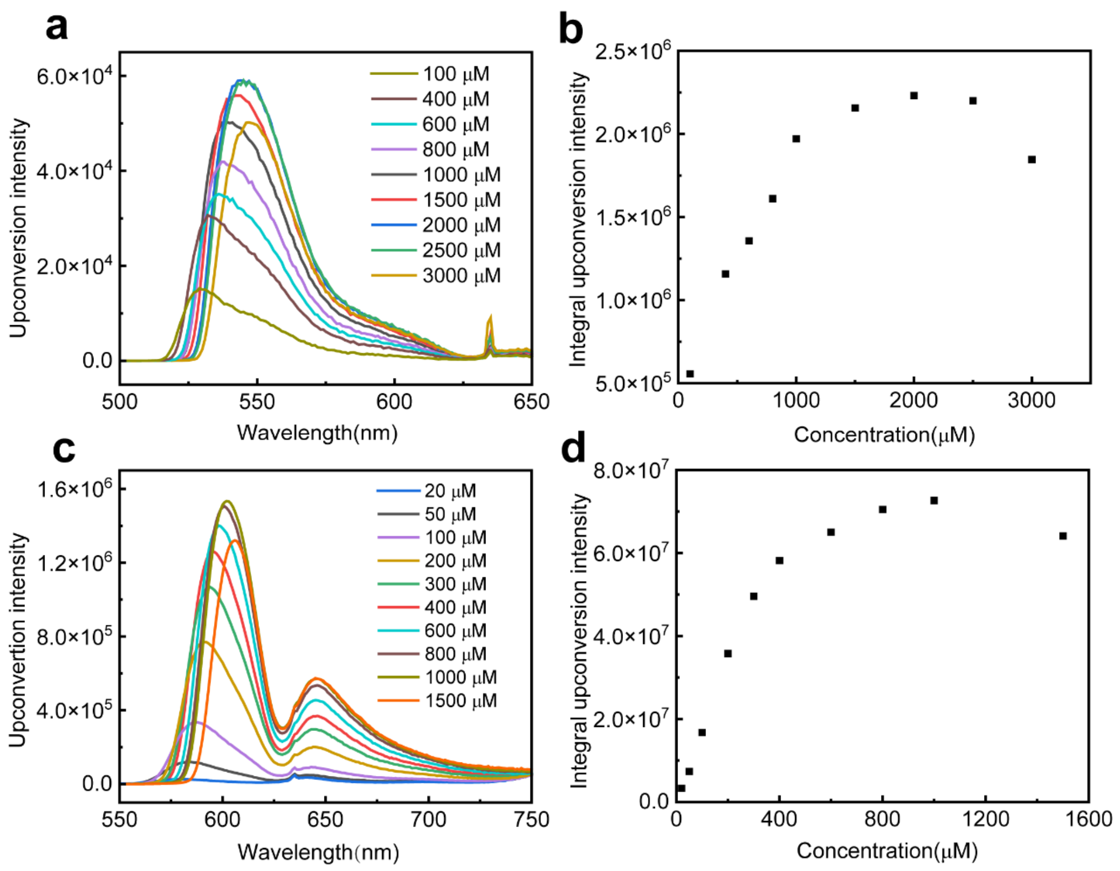

Figure S10. (a) The upconversion emission spectra of PdTPBP (10 μM) and different concentrations of B-1 in degassed toluene; (b) quantitative analysis of the relationship between upconversion intensity and concentration of B-1. λ_{ex} = 635 nm, 318 mW/cm^2. (c) The upconversion emission spectra of PdTPBP (10 μM) and different concentrations of B-2 in degassed toluene; (d) quantitative analysis of the relationship between upconversion intensity and concentration of B-2. λ_{ex} = 635 nm, 196 mW/cm^2.

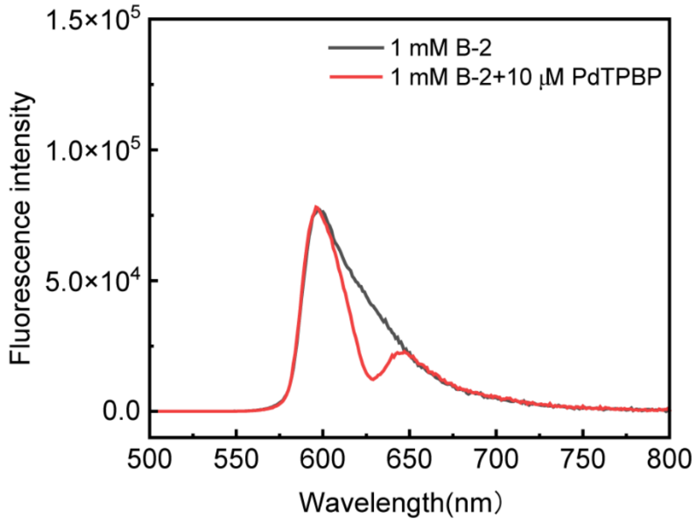

Figure S11. Fluorescence spectra of B-2 (1 mM) with or without photosensitizer (10 μM), λ_{ex} =470 nm.

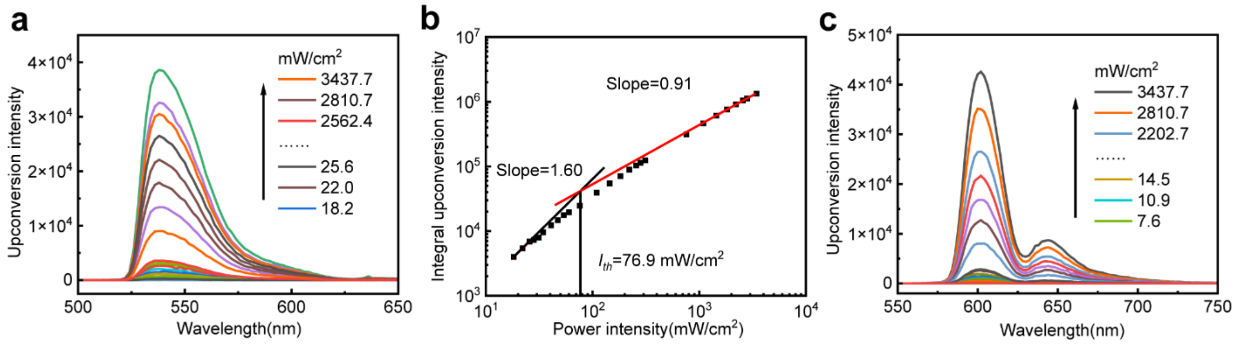

Figure S12. (a) Incident light power dependence study of TTA-upconversion for PdTPBP (10 μM) and B-1 (1000 μM) in degassed toluene. (b) The double-logarithmic plot of B-1 integrated emission intensity was drawn as a function of 635 nm excitation power density. Solid lines illustrate a slope of 1.60 (black, quadratic) and a slope of 0.93 (red, linear), and I_{th} is 65.3 mW/cm². (c) Incident light power dependence study of TTA-upconversion for PdTPBP (10 μM) and B-2 (1000 μM) in degassed toluene.

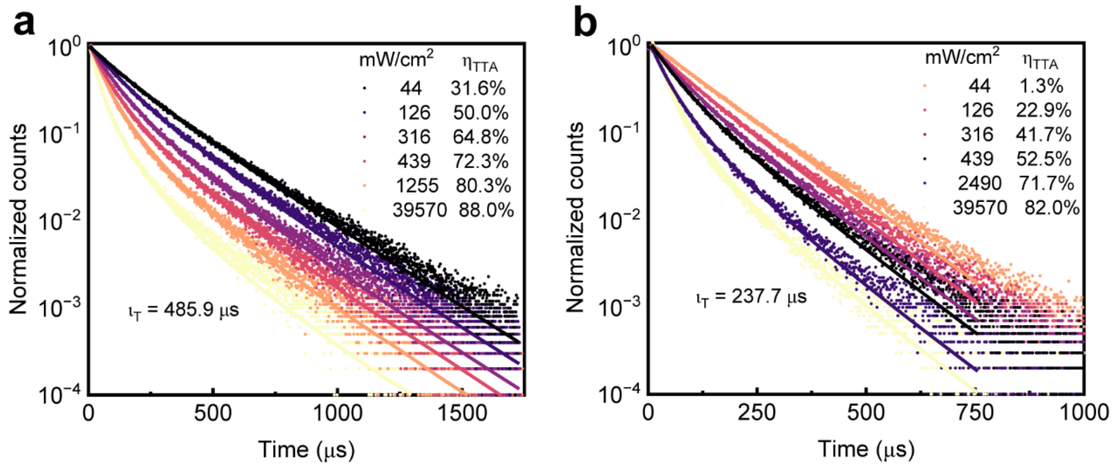

Figure S13. η_{TTA} determination of annihilator compounds. (a) B-1, (b) B-2.

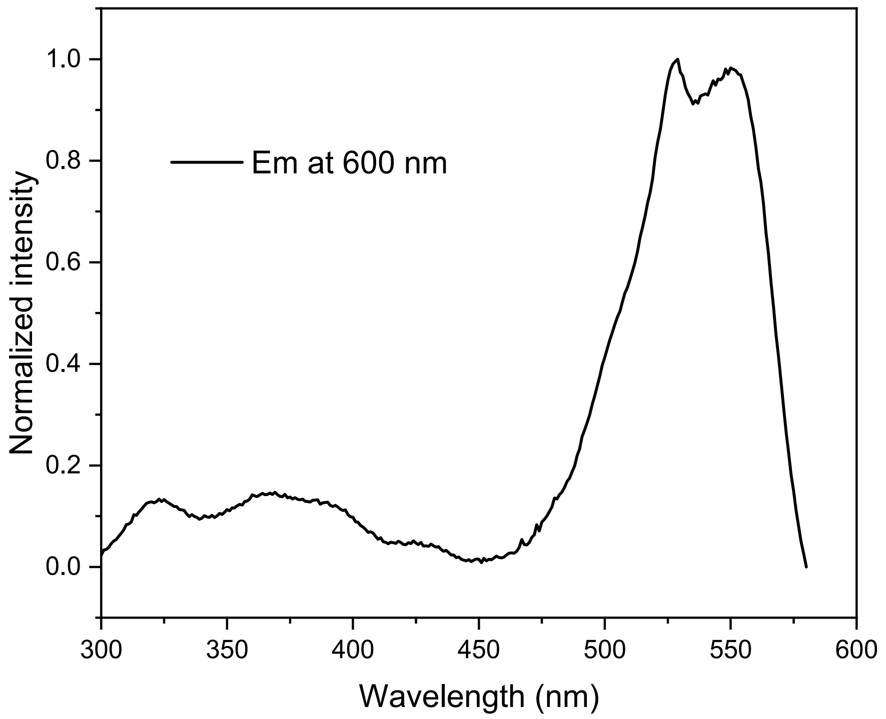

Figure S14. The excitation spectrum of B-2 in toluene, λ_{em} = 600 nm.

Table S1. Density functional theory (DFT) calculation for the annihilators. The electronic excitation energies (eV) and corresponding oscillator strengths (*f*), main configurations, and CI coefficients of the low-lying electronic excited states of compound B-1 and B-2.

**Compound**	**Electronic transition** [a]	**Energy** [b] **(eV)**	**Wavelength (nm)**	f^c	**Composition** [d]	**CI**[e]
B-2	$S_0 \rightarrow S_1$	2.54	487	0.1839	H→L+1	0.63331
	$S_0 \rightarrow S_2$	2.56	485	0.0016	H→L	0.59008
	$S_0 \rightarrow T_1$	1.52	816	0.0000 [g]	H→L	0.52537
	$S_0 \rightarrow T_2$	1.53	812	0.0000 [g]	H→L+1	0.50904
B-1	$S_0 \rightarrow S_1$	2.92	425	0.6000	H→L	0.70221
	$S_0 \rightarrow S_2$	3.50	354	0.0733	H-1→L	0.69584
	$S_0 \rightarrow T_1$	1.53	810	0.0000 [g]	H→L	0.71033
	$S_0 \rightarrow T_2$	2.73	455	0.0000 [g]	H-1→L	0.69054

[a] Vertical excitation energy from the ground state to the excited state was

calculated by TD-DFT//B3LYP/6-31G(d), based on the DFT//B3LYP/6-31G(d)-optimized ground state geometries. [b] Only the selected low-lying excited states are presented. [c] Oscillator strengths. [d] TD-DFT//B3LYP/6-31G(d)-optimized excited state geometries. [e] CI coefficients are in absolute values. [f] No spin–orbital coupling effect was considered; thus, the f values are zero.

Table S2. S_1 and T_1 energy levels of B-1, B-2, obtained by experiments, CAM-B3LYP, and B3LYP calculations.

	Exp. S_1	CAM-B3LYP S_1	B3LYP T_1	CAM-B3LYP T_1
B-1	2.43 eV	2.8634 eV	1.53 eV	1.2476 eV
B-2	2.21 eV	2.6970 eV	1.52 eV	1.2464 eV

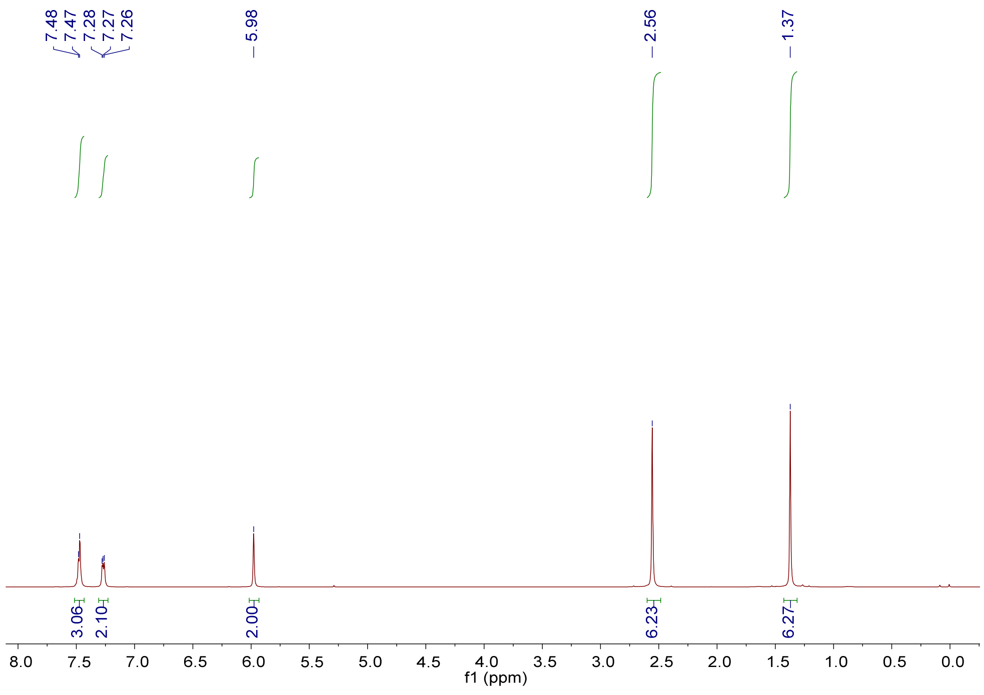

Figure S15. ^{1}H-NMR (400 MHz, $CDCl_3$) of B-1.

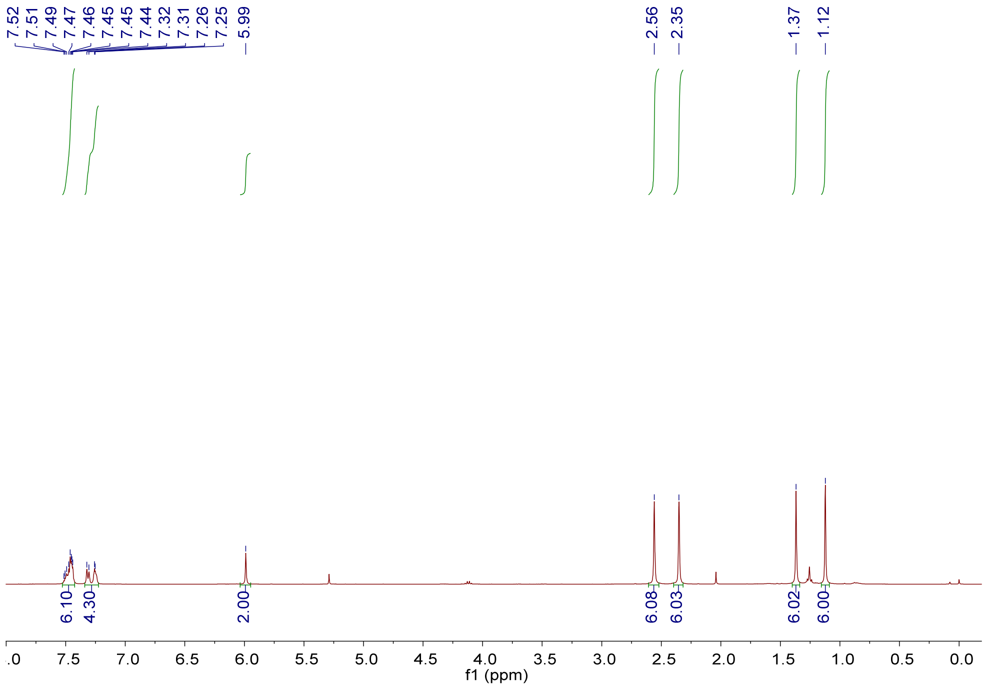

Figure S16. ^{1}H-NMR (400 MHz, $CDCl_3$) of B-2.

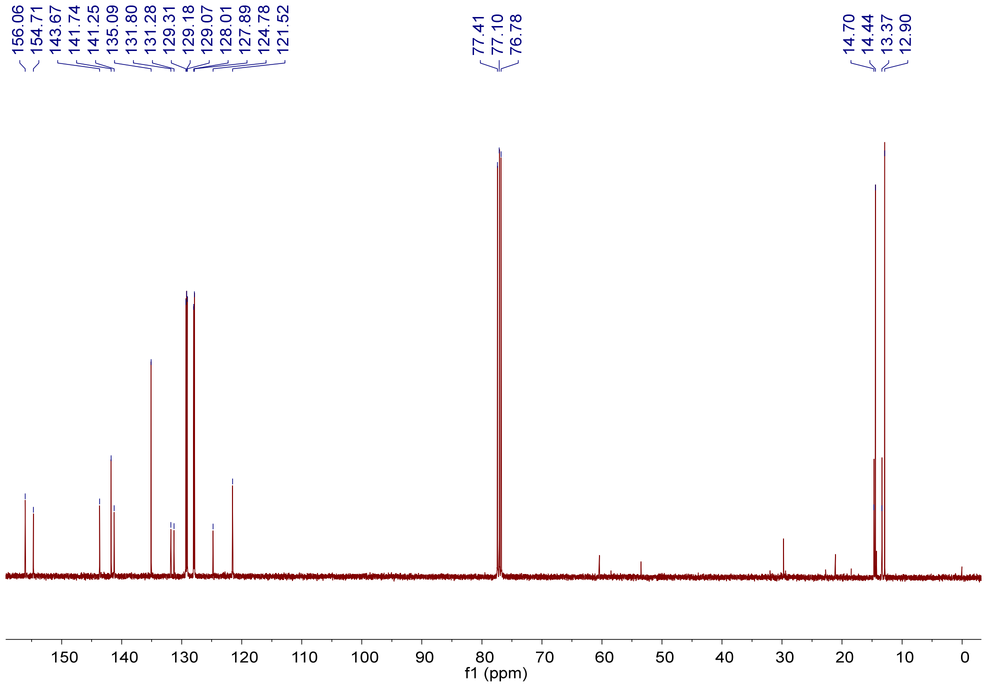

Figure S17. ^{13}C-NMR (100 MHz, $CDCl_3$) of B-2.

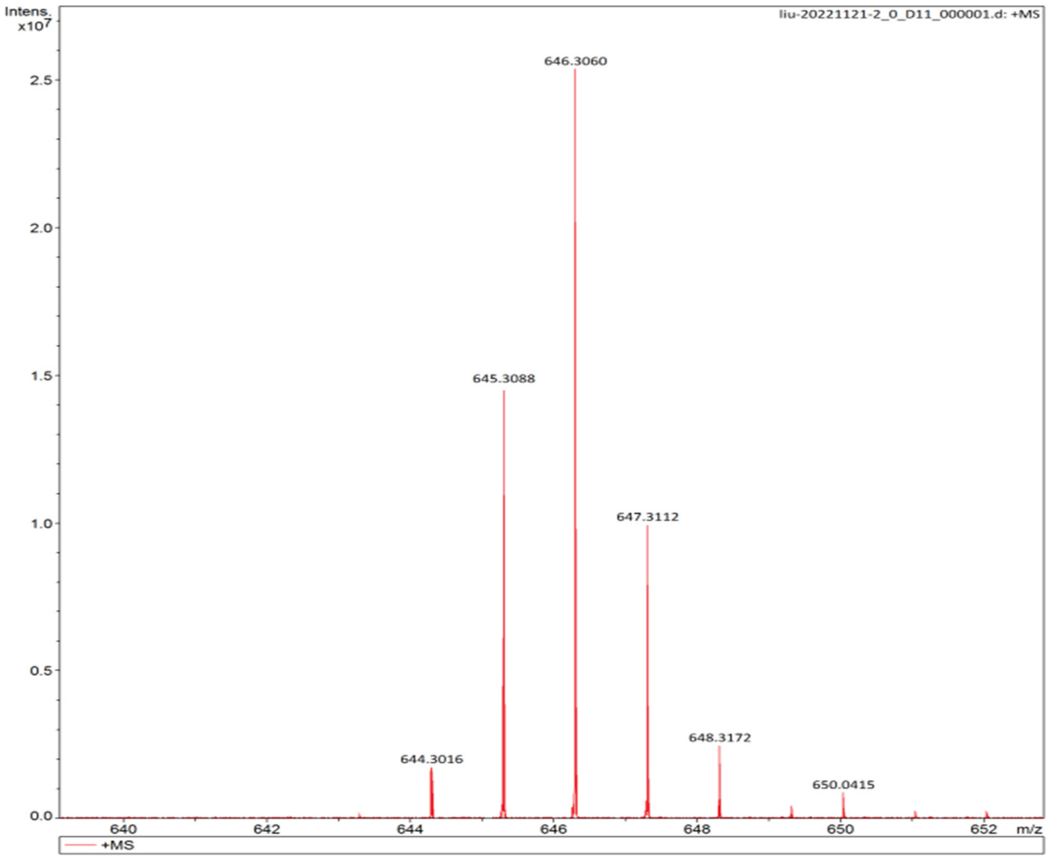

Figure S18. HRMS (MALDI) of B-2.

Reference

1. Mu, Y.-d.; Guo, D.-c.; Hou, Y.-j.; Wang, S.-h., Facile electrospinning preparation and superior luminescence properties of BODIPY composite nanofibers. *Text. Res. J.* **2016,** 87, 1795-1805.
2. Hayashi , Y.; Yamaguchi , S.; Cha, W. Y.; Kim, D. ; Shinokubo, H, Synthesis of Directly Connected BODIPY Oligomers through Suzuki-Miyaura Coupling. *Org. Lett.* **2011,** 13, 2992-2995.
3. Nepomnyashchii, A. B.; Broring, M.; Ahrens, J.; Bard, A. J., Synthesis, photophysical, electrochemical, and electrogenerated chemiluminescence studies. Multiple sequential electron transfers in BODIPY monomers, dimers, trimers, and polymer. *J. Am. Chem. Soc.* **2011,** 133, 8633-8645.
4. Singh, V. K.; Yu, C.; Badgujar, S.; Kim, Y.; Kwon, Y.; Kim, D.; Lee, J.; Akhter, T.; Thangavel, G.; Park, L. S.; Lee, J.; Nandajan, P. C.; Wannemacher, R.; Milián-Medina, B.; Lüer, L.; Kim, K. S.; Gierschner, J.; Kwon, M. S., Highly efficient organic photocatalysts discovered via a computer-aided-design strategy for visible-light-driven atom transfer radical polymerization. *Nat. Catal.* **2018,** 1, 794-804.
5. Deng, F.; Sun, W.; Castellano, F. N., Texaphyrin sensitized near-IR-to-visible photon upconversion. *Photochem. Photobiol. Sci.* **2014,** 13, 813-9.
6. Mattiello, S.; Monguzzi, A.; Pedrini, J.; Sassi, M.; Villa, C.; Torrente, Y.; Marotta, R.; Meinardi, F.; Beverina, L., Self-Assembled Dual Dye-Doped Nanosized Micelles for High-Contrast Up-Conversion Bioimaging. *Adv. Funct. Mater.* **2016,** 26, 8447-8454.
7. Kwon, T.,-H; Kim, M. K.; Kwon,J.; Shin, D.-Y; Park, S. J.; Lee, C.-Y; Kim, J.-J; Hong, J.-I, Highly Efficient Light-Harvesting System Based on a Phosphorescent Acceptor Coupled with Dendrimer Donors via Singlet-Singlet and Triplet-Triplet Energy Transfer. *Chem. Mater.* **2007,** 19, 3673-3680.
8. Olesund, A.; Johnsson, J.; Edhborg, F.; Ghasemi, S.; Moth-Poulsen, K.; Albinsson, B., Approaching the Spin-Statistical Limit in Visible-to-Ultraviolet Photon Upconversion. *J. Am. Chem. Soc.* **2022,** 144, 3706-3716.
9. Li, J. K.; Zhang, M. Y.; Zeng, L.; Huang, L.; Wang, X. Y., NIR-Absorbing B,N-Heteroarene as Photosensitizer for High-Performance NIR-to-Blue Triplet-Triplet Annihilation Upconversion. *Angew. Chem. Int. Ed.* **2023,** 62, e202303093.
